# Role of Mitochondria in Neurodegenerative Diseases: From an Epigenetic Perspective

**DOI:** 10.3389/fcell.2021.688789

**Published:** 2021-08-27

**Authors:** Sutong Xu, Xi Zhang, Chenming Liu, Qiulu Liu, Huazhen Chai, Yuping Luo, Siguang Li

**Affiliations:** ^1^Key Laboratory of Spine and Spinal Cord Injury Repair and Regeneration of Ministry of Education, Orthopedic Department of Tongji Hospital, Tongji University School of Medicine, Shanghai, China; ^2^Stem Cell Translational Research Center, Tongji Hospital, Tongji University School of Medicine, Shanghai, China

**Keywords:** mitochondria, metabolism, mtDNA, epigenetics, neurodegenerative diseases

## Abstract

Mitochondria, the centers of energy metabolism, have been shown to participate in epigenetic regulation of neurodegenerative diseases. Epigenetic modification of nuclear genes encoding mitochondrial proteins has an impact on mitochondria homeostasis, including mitochondrial biogenesis, and quality, which plays role in the pathogenesis of neurodegenerative diseases like Alzheimer’s disease, Parkinson’s disease, Huntington’s disease, and amyotrophic lateral sclerosis. On the other hand, intermediate metabolites regulated by mitochondria such as acetyl-CoA and NAD^+^, in turn, may regulate nuclear epigenome as the substrate for acetylation and a cofactor of deacetylation, respectively. Thus, mitochondria are involved in epigenetic regulation through bidirectional communication between mitochondria and nuclear, which may provide a new strategy for neurodegenerative diseases treatment. In addition, emerging evidence has suggested that the abnormal modification of mitochondria DNA contributes to disease development through mitochondria dysfunction. In this review, we provide an overview of how mitochondria are involved in epigenetic regulation and discuss the mechanisms of mitochondria in regulation of neurodegenerative diseases from epigenetic perspective.

## Introduction

**FIGURE 1:**
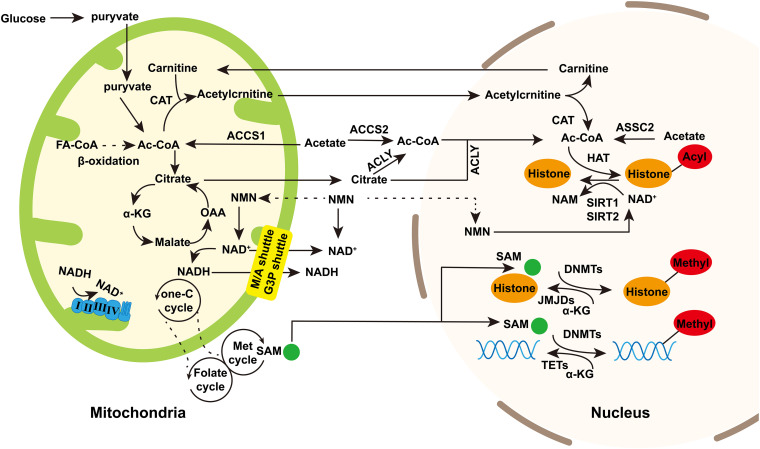
The regulation of mitochondrial intermediate metabolites to nuclear epigenetics. Mitochondria provide substrates for nuclear epigenetics such as DNA or histone methylation and histone acetylation/deacetylation. Acetyl-CoA is the source of acetyl groups used in histone acetylation. It is produced during fatty acid synthesis and glycolysis. Besides, acetate is catalyzed by ASSC1 in mitochondria and ASSC2 in cytoplasm and nucleus to generate acetyl-CoA. Acetyl-CoA in the nucleus and cytoplasm can be exchanged through nuclear pores. Acetyl-CoA enters into TCA cycle to produce citrate in mitochondria which is transported into cytoplasm and then catalyzed by ACLY to regenerate acetyl-CoA. Acetyl-CoA produced in mitochondria can also be regenerated in nucleus trough carnitine/acylcarnitine translocator. NAD^+^ is the cofactor of sirtuins in deacetylation. In TCA cycle, NAD^+^ is converted into NADH, and then NADH reconverted into NAD^+^ by complex I and III in OXPHOS. NAD^+^/NADH in mitochondria can be exchanged in cytoplasm and mitochondria through malate/aspartic acid shuttle and glyceraldehyde-3-phosphate shuttle. NAM is potentially transported into mitochondria and nucleus to generate NAD^+^. *S*-adenosine methionine (SAM) is generated through the coupling of the folate and methionine cycles in the cytosol and sustained by the one-carbon (one-C) metabolism in mitochondria. SAM can enter into nucleus as the source of methyl group in histone or DNA methylation. α-Ketoglutarate (α-KG), the intermediate of TCA cycle, can enter into nucleus as the cofactor of JMJDs and histone demethylases and TET DNA demethylases. Dashed arrows indicate potentially indirect connections or pathways that need further validation. Abbreviations: FA-CoA, fatty acyl CoA; ac-CoA, acetyl-CoA; CAT, carnitine acetyltransferase; OAA, oxaloacetic acid; ACLY, ATP-citrate lyase; NMN, nicotinamide mononucleotide; M/A shuttle, malate/aspartate shuttle; and G3P shuttle, glyceraldehyde 3-phosphate shuttle.

Mitochondria originated from *Alphaproteobacteria* with a circular genome packaged into DNA–protein assemblies. The mammalian mitochondrial genome is 16.6 kb and encodes 37 genes, including 2 ribosomal RNAs (12S and 16SrRNA), 22 tRNAs, and 13 core component proteins of the respiratory electron transport chain (ETC). Some miRNAs and lncRNAs are found transcript from mitochondria DNA (mtDNA; Mercer et al., 2011; Rackham et al., 2011). Mitochondrial function is regulated by both nuclear and mitochondrial genome, since most of proteins in mitochondria are encoded by nuclear and transported into mitochondria leading by mitochondrial targeting sequences. Indeed, next to producing energy, other mitochondrial functions have come into focus. Mitochondria are involved in metabolic processes and modulate several signal transduction pathways. The crosstalk between nucleus and mitochondria is identified as bidirectional micronucleus communication, which is essential for maintaining cell homeostasis and shaping diseases (Quirós et al., 2016; Mottis et al., 2019). Specially, the micronucleus communication is involved in epigenetic regulation (Bellizzi et al., 2012; Quirós et al., 2016). Epigenetic regulation, responding to environmental stimulation to regulate gene expression without changing the genome, is primarily divided into DNA methylation, histone post-translational modification (acetylation, methylation, etc.), chromatin open state and non-coding RNA regulation. Nuclear epigenome can drive changes in mitochondria functions, and in turn, signaling molecules mediated by metabolites can travel from mitochondria to nucleus which may play a role in nuclear epigenetic regulation. Mitochondrial metabolic intermediates, such as acetyl-CoA and NAD^+^, are the substrate for acetylation and a cofactor of deacetylation, respectively, (Figure 1). *S*-adenosine methionine (SAM) is involved in both DNA and histone methylation. It is produced through the coupling of folate and methionine cycles in the cytoplasm, which is maintained by one-carbon (One-C) metabolism in mitochondria (Figure 1). Moreover, α-Ketoglutarate, a key intermediate in the tricarboxylic acid (TCA) cycle of oxidative phosphorylation (OXPHOS), is required for Jumonji C domain demethylases and DNA demethylase translocation (TET) as cofactors (Xu et al., 2011; Figure 1). These intermediates may directly or indirectly provide by mitochondria and affect the epigenetic regulation of the nuclear genome. In addition, although the mitochondrial genome lacks histones, mtDNA can be modified by methyltransferase to participate in epigenetic regulation. In recent years, there has been an increasing interest in mtDNA methylation in neurodegenerative diseases.

Mitochondria are bimembranous organelles whose inner membranes fold inwards into a crista-like structure and contain five enzyme complexes OXPHOS (complex I–V). They synthesize ATP through the ETC, which supports the biosynthesis and metabolic demand of cells. Normal integrity and function are very important for the maintenance of cell vitality. Mitochondria are dynamic organelles that can adapt to physiological changes by changing their morphology or number. A number of studies have shown the importance of mitochondrial dysfunction in the pathogenesis of neurodegenerative diseases such as Alzheimer’s disease (AD), Parkinson’s disease (PD), amyotrophic lateral sclerosis (ALS), and Huntington’s disease (HD; Lin and Beal, 2006; Yao et al., 2009; Chaturvedi and Flint Beal, 2013). In these diseases, mitochondria are characterized by decreased activity of respiratory chain enzyme, abnormal morphology, and more mutations in mtDNA (Chaturvedi and Flint Beal, 2013). Regulating mitochondrial function or changing mitochondrial metabolites is a strategy to treat neurodegenerative diseases.

This review will focus on the role of the mitochondria in the epigenetic regulation of neurodegenerative diseases, including how nuclear epigenome affects mitochondrial function and mitochondrial epigenetic regulation.

## Mitochondria Are Involved in the Pathogenesis of Several Neurodegenerative Diseases

### The Maintenance of Mitochondrial Homeostasis Determines the Normal Function of Mitochondria

As energy supply station, mitochondria maintain their homeostasis and keep functional in many ways, including mitochondrial biogenesis, mitochondrial dynamics, mitophagy, and apoptosis.

**FIGURE 2:**
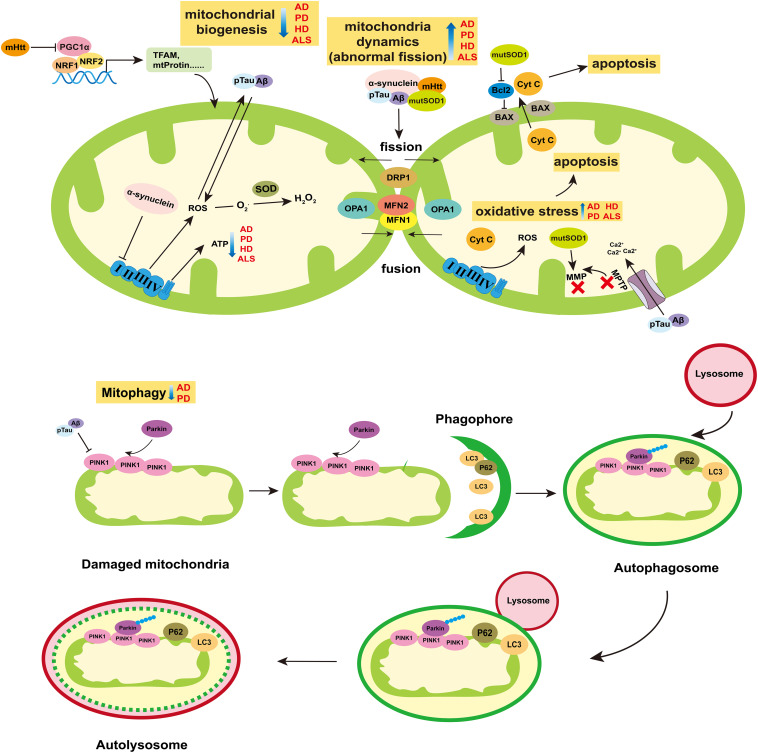
Role of mitochondria in neurodegenerative diseases. The breakdown of mitochondrial homeostasis is considered to play crucial role in the pathogenesis of neurodegenerative diseases. Mitochondrial biogenesis, dynamics, mitophagy, and ROS-mediated cell apoptosis are ways for mitochondria to maintain homeostasis. The ability of mitochondrial biogenesis is reduced in AD, PD, HD, and ALS. Particularly, the mHtt can suppress the activity of PGC1α, which is the main cause of mitochondria dysfunction in HD. Abnormal mitochondrial division appear in AD, PD, HD, and ALS. The pTau and Aβ of AD, α-synuclein of PD, mHtt of HD and mutSOD of ALS can increase the protein levels of mitochondria fission (DRP1), and reduce mitochondria fusion (MFN1/2 and OPA1). The release of ROS increases in those four diseases, which can induce oxidative stress and then mediate cell apoptosis. In AD and PD, mitophagy is blocked, which prevents the damaged mitochondria from being removed effectively. Moreover, α-synuclein can inhibit the activity of complex I of ETC. pTau and Aβ can enlarge the MPTP to decrease the MMP, which can reduce the synthesis of ATP. Besides, the mutSOD can destroy the MMP and have impair on mitochondria functions. Abbreviations: AD, Alzheimer’s disease; PD, Parkinson’s disease; HD, Huntington’s disease; ALS, amyotrophic lateral sclerosis; Aβ, β-Amyloid protein; pTau, phosphorylated Tau; mHtt, mutation Htt protein; mutSOD, mutation SOD protein; MMP, mitochondrial membrane potential; and MPTP, Mitochondrial Permeability Transition Pore.

Mitochondria biogenesis is the progress that new mitochondria are producted from existing one. In mammals, mitochondrial transcription begins at D-Loop region where the mitochondrial RNA polymerase (POLRMT) and the mitochondrial transcription factor A (TFAM) interact directly to bind transcription start site to regulate mtDNA transcription. Mitochondrial transcription factor B2 is a key factor assisting POLRMT in promoting mitochondrial RNA transcription initiation. Transcriptional termination is then regulated by Mitochondrial Transcription Termination Factor 1. In addition, the proliferator-activated receptor γ coactivator-1 (PGC1) family (PGC1-α and PGC1-β) and several transcription factors subsequently regulated by PGC1-related cofactor (PRC), including NRF1, NRF2, and YIN-YANG 1 (YY1), affect mitochondrial gene expression (Vercauteren et al., 2006, 2008, 2009; Cunningham et al., 2007; Shao et al., 2010; Figure 2).

Mitochondria are highly dynamic organelle undergo continuous fusion and fission events to keep their shape and size normal, which is mediated by GTPases, including dleprotein-like protein 1 (DRP1), mitochondrial fusion protein 1 (MFN1), mitochondrial fusion protein 2 (MFN2), and optic nerve atrophy protein 1 (OPA1). MFN1 and MFN2 are transmitochondrial extramembrane proteins that regulate mitochondria fusion, while DRP1 remains in the cytoplasm until it is recruited to outer mitochondrial membrane (OMM) for fission (Koshiba et al., 2004; Figure 2). OPA1 is in the inner mitochondrial membrane (IMM) and plays a role in balancing the fission and fusion of mitochondria (Friedman and Nunnari, 2014; Figure 2).

Mitophagy, another form of mitochondrial quality maintenance, is mainly mediated by PINK1 and Parkin (Figure 2). PINK1 is a constitutive circulating kinase on the OMM, and Parkin is an E3 ubiquitin ligase which can be phosphorylated at Ser65 in UBL domain by PINK1 and recruited to damaged mitochondria to polyubiquitinates its substrates (Pickrell and Youle, 2015). Autophagy receptor proteins such as SQSTM1/P62 combined with LC3, bind ubiquitinated cargo and connect them to the autophagosome, then the autophagosome is eliminated by lysosome (Pickrell and Youle, 2015).

Reactive oxygen species (ROS)-mediated mitochondrial oxidative stress damage can induce cell apoptosis. ROS such as superoxide anion (O2•-), hydroxyl radical (OH•), and stable hydrogen peroxide (H_2_O_2_) mainly come from ETC where some H^+^ leak from the complex I and III and react with dioxygen (Murphy, 2009). The superoxide dismutases (SODs) family, including the mitochondrial MnSOD (SOD2) and the intracellular (SOD1) or extracellular (ECSOD, SOD3) isoforms of Cu/ZnSOD, catalyze the superoxide anions into oxygen and hydrogen peroxide. Then, hydrogen peroxide is converted into water by glutathione peroxidase and catalase, acting as an additional antioxidant defense (Lubos et al., 2011; Dimauro et al., 2020). The imbalance between ROS and the antioxidant defense system causes oxidative stress damage, which is one of the causes of mitochondrial dysfunction and cell apoptosis.

### Mitochondria Dysfunction Is Crucial for the Pathogenesis of Neurodegenerative Diseases

Neurodegenerative diseases are complex diseases regulated by heredity and environment, which are characterized by a gradual loss of neuronal function and the gradual deterioration with the progress of neurodegenerative diseases. Imbalance of mitochondrial homeostasis is present in AD, PD, HD, and ALS, which is shown in Figure 2.

Alzheimer’s disease characterized by a decline in cognitive function is the most common cause of dementia (Battle, 2013). It is divided into early-onset familiar AD and late-onset sporadic AD. The accumulation of beta-amyloid (Aβ) and phosphorylated Tau (pTau) in the brain is a pathological feature of AD (Hyman et al., 2012). Aβ and pTau affect mitochondrial integrity and aggravate mitochondrial dysfunction (Tönnies and Trushina, 2017). Meanwhile, damaged mitochondria induce excessive oxidative stress which promotes the accumulation of these two proteins in turn (Tönnies and Trushina, 2017). The oxidative damage induced by Aβ and pTau leads to the decrease protein levels of PINK1 and Parkin, which inhibits mitochondrial autophagy, thereby increasing the amount of Aβ and pTau (Kerr et al., 2017). Aβ and pTau induce the enlargement of mitochondrial permeability transition pore (mPTP), destroy the permeation barrier of IMM, and lead to the decrease of intermembrane potential (Szeto, 2014; Quntanilla and Tapia-Monsalves, 2020). At the same time, cyt C and other pro-apoptotic proteins escape into the cytoplasm through mPTP and trigger cell apoptosis and death (Szeto, 2014). In addition, abnormal mitochondrial fission and decreased expression of mitochondrial biogenesis-related proteins (PGC-1α, TFAM, and NRF2) were found in AD patients, AD mouse models and AD cell models, which indicates that mitochondria dynamic and biogenesis are impaired (Manczak et al., 2011; Rice et al., 2014). On the other hand, mutations in amyloid precursor protein (APP), presenilin-1 (PS1), and presenilin-2 loci contribute to the early onset of familial AD, which is considered to predispose to greater mitochondrial dysfunction (Manczak et al., 2011; Hyman et al., 2012; Rice et al., 2014).

Parkinson’s disease is characterized by cardinal motor manifestations of tremor and bradykinesia, the signature clinical manifestation of which is a decrease in dopamine neurons and dopamine level in the substantia nigra pars compacta (Samii et al., 2004). It is divided into familiar PD and sporadic PD. The causes of PD include mitochondrial dysfunction, abnormal protein aggregation, chronic inflammation, and oxidative imbalance (Subramaniam and Chesselet, 2013; Bose and Beal, 2016; Rocha et al., 2018). Reduced activity of ETC complex I is found in PD substantia nigra (SN). Interestingly, the neurotoxin 1-Methyl-4-phenyl-1,2,3,6-tetrahydropyridine (MPTP), which targets mitochondrial complex I, induces animal phenotypes similar to PD (Ballard et al., 1985). In dopamine neurons, mitochondrial dysfunction is an important cause of oxidative stress which plays an important role in the production of neurotoxins. Besides, α-synuclein aggregates to form Lewy bodies or Lewy neurites, which are also one of the pathological features of PD (Gorbatyuk et al., 2008; Volpicelli-Daley et al., 2011, 2014). The accumulation of α-synuclein in the outer membrane may interfere with the protein import mechanism (Rocha et al., 2018). And growing evidence show that α-synuclein can affect mitochondrial dynamics, particularly by disrupting the mitochondrial fusion (Kamp et al., 2010; Nakamura et al., 2011; Fabian and Sonenberg, 2012). In addition, there are several pathogenic genes of familial PD. For example, SCNA and LRRK2 control autosomal dominant PD, while Parkin, PINK1 and ATP13A2 control autosomal recessive PD.

Huntington’s disease is characterized by the presence of aggregation of Huntington mutated protein (mHtt), and the main clinical symptoms of HD are cognitive decline, progressive dyslexia, and psychiatric disorders. MHtt binds to DRP1 to trigger mitochondrial abnormal fission. The mRNA levels of DRP1 and FIS1 increase with the progression of HD, but the expression of MFN1/2 does the opposite (Kim et al., 2010; Shirendeb et al., 2011; Song et al., 2011). MHtt interacts with autophagy receptors, preventing them from binding to damaged mitochondria and preventing autophagosomes from engorging abnormal mitochondria (Martinez-Vicente et al., 2010). Particularly, mHtt can inhibit PGC-1α and reduce mitochondrial biogenesis, which is a common root cause of mitochondrial dysfunction in HD (Chaturvedi et al., 2009; Johri et al., 2013).

Amyotrophic lateral sclerosis is characterized by progressive neurodegeneration in brain and spinal cord (Kwong et al., 2006). Oxidative stress caused by the production and accumulation of ROS is one of the main factors of ALS pathology (Cozzolino et al., 2008). Mutation of Cu/Zn SOD1 gene is the main manifestation of ALS (Rosen, 1993). The accumulation of mutated SOD (mutSOD) protein in mitochondria may cause mitochondrial dysfunction through a variety of ways. It damages the mitochondrial membrane, resulting in reduced mitochondrial membrane potential (MMP), mitochondrial swelling, and vacuolar degeneration (Wong et al., 1995; Kong and Xu, 1998). The mutSOD binds to the apoptotic regulator Bcl-2 to alter the electrical conductivity of voltage dependent anionchannel, reduce ATP production, and increase calcium accumulation (Guégan et al., 2001; Takeuchi et al., 2002; Kirkinezos et al., 2005; Ferri et al., 2006). Formation of the toxic mutSOD1/Bcl-2 complex leads to conformational changes in Bcl-2, as well as mitochondrial dysfunction, including altered mitochondrial morphology, disruption of mitochondrial membrane integrity, and increased release of cyt C (Pedrini et al., 2010). Moreover, expression of mutSOD1 in neurons decreased the expression level of OPA1 and increased the level of phosphorylated DRP1, which induces abnormal mitochondrial fragmentation (Ferri et al., 2010).

## Mitochondria Are Involved in Nuclear Epigenetic Regulation in Neurodegenerative Diseases

DNA methylation means that DNA is methylated by adding a methyl group at the 5′ position of the cytosine to produce 5-methylcytosine (5mC), which usually occurs at cytosine-phosphate-guanine (CpG) islands. The key step of methylation is mediated by three DNA methyltransferases (DNMT): DNMT1, DNMT3A, and DNMT3B. DNMT1 preferentially methylates hemimethylated DNA and maintains genomic DNA methylation patterns following DNA replication, while DNMT3A and DNMT3B have the responsible to *de novo* methylation of DNA (Pradhan et al., 1999; Ramsahoye et al., 2000).

Data from several studies suggest that nuclear genomic DNA methylation can affect the development of PD by regulating mitochondria functions. Previous studies have shown no PARK2 (Parkin) differential DNA methylation in the brains of PD patients and healthy controls (De Mena et al., 2013). Recently, the methylation levels of parkin promoter were significantly reduced in early-onset Parkinson’s disease patients shown in the epigenome-wide association study (Eryilmaz et al., 2017). Moreover, in SN of sporadic PD, DNA hypermethylation was found in the promoter of PGC-1α, an important transcription factor that regulates the mitochondria biological functions (Su et al., 2015).

Mitochondria can indirectly regulate the production of SAM and affect the nuclear epigenome (Figure 1). SAM, a methionine metabolite, is a source of methyl groups used in the nucleus for histone and DNMT. One-C metabolism includes the folate cycle and the methionine cycle. 5-methyl-THF (5-MTHF), an intermediate product of the folate cycle in the cytoplasm, participates in methylation of homocysteine to methionine, which enters the methionine cycle to produce SAM (Ormazabal et al., 2015). The connection between mitochondria and cytoplasm is through the exchange of One-C donors such as serine and glycine (Tibbetts and Appling, 2010). Mitochondria are unable to synthesize SAM due to lack of methionine adenosyltransferase activity, so the SAM synthesized in cytoplasmic is transferred into mitochondria through methionine carriers/transporters (Agrimi et al., 2004). The mitochondrial One-C cycle and ATP maintain the synthesis of SAM in the cytoplasm. SAM is used by DNMTs and then it converted into *S*-adenosine homocysteine (SAH; Chiang et al., 1996). SAH rapidly hydrolyzes to Homocysteine (Hcy), which is then catabolized or remethylated to methionine (Medina et al., 2001). Changes in carbon metabolism can directly affect DNA methylation through SAM and SAH levels and regulation of methyltransferase activity. Late-onset AD is associated with hyperhomocysteinemia which is influenced by diet (vitamin B6, vitamin B12, and folic acid; Fuso et al., 2008). Vitamin B deficiency induced the accumulation of Hcy and SAH, thus impeding methyltransferase activity (Fuso et al., 2011). Specific demethylation of PS1 promoter sites resulted in overexpression of PS1 and upregulation of γ-secretase activity, which might promote overproduction of Aβ (Li et al., 2015). Folic acid deficiency increased the protein levels of APP, PS1, and Aβ proteins in hippocampus (Li et al., 2015). After folic acid supplementation, APP and PS1 promoter methylation rates increased and APP, PS1, and Aβ protein levels decreased (Li et al., 2015).

## Mitochondria Genome Methylation Is Involved in Neurodegenerative Diseases

### Methyltransferases Are Diverse in Mitochondria

The present of mtDNA methylation is controversial because of imperfect detection methods (van der Wijst and Rots, 2015). Although the earliest studies reported that mtDNA was not methylated, subsequent studies found low levels of CpG dinucleotide methylation in mitochondria of several species (Nass, 1973; Number et al., 1983; Pollack et al., 1984). In human colon carcinoma cells (HCT116) and mouse embryonic fibroblasts cells (MEFs), DNMT1 transcriptional variants were found to translocated to mitochondria driven by mitochondrial targeting sequences and bound to a unique non-coding region called D-Loop (Shock et al., 2011). D-Loop is the start of mitochondrial DNA replication and transcription, where has two promoters, namely heavy chain promoter (HSP) and light chain promoter. DNMT3A was not found in MEFs and HCT116 cell lines, however, according to further studies, it was found in motor neurons (Chestnut et al., 2011; Shock et al., 2011). It indicates that these methyltransferases in mitochondria may be tissue specific. In addition, DNMT1 and low expression level of DNMT3B were observed in the mitochondria of mouse 3T3-L1 and HeLa cells, and inactivation of these two transmethylase could reduce methylation levels at CpG site (Bellizzi et al., 2013).

The demethylation pathway may play a role in mitochondria. One of DNA demethylation mechanisms is initiated by the oxidation of 5mC to 5-hydroxymethylcytosine (5hmC) by ten-eleven translocation (TET) enzymes (Ito et al., 2011). Both TET1 and TET2 are showed in the mitochondria, along with the presence of 5hmC in the D-Loop (Bellizzi et al., 2013). Additionally, evidence presented that 5mC and 5hmC existed stably at cytosine without guanine base in mtDNA, suggesting the role of non-CpG methylation in mtDNA (Bellizzi et al., 2013). CpG and non-CpG methylation sites are in HSP promoter regions and conserved sequence blocks, thus the epigenetic modifications may adjust mtDNA copy and transcription (Ito et al., 2011).

### The Extent of mtDNA Methylation Varies Among Neurodegenerative Diseases Compared With Controls

The methylation level of mtDNA changes dynamically with the development of AD. Initial dot blot analysis showed that mitochondrial 5hmC levels in superior and middle temporal gyrus of preclinical AD and late-stage AD subjects were elevated, but it remained further verification due to the small number of samples used (Bradley-Whitman and Lovell, 2013). A later study found that mitochondrial 5mC levels in D-Loop region of mtDNA in the entorhinal cortex were increased in human postmortem brains with AD-related pathology (stages I to II and stages III to IV of Braak and Braak) compared with control cases, and the methylation levels were higher in early stages (stages I/II) than in later stages (stages III/IV; Blanch et al., 2016). Whereas, another study showed the mitochondrial D-Loop methylation levels in peripheral blood of late-onset Alzheimer’s disease patients were significantly lower than those of healthy controls (Stoccoro et al., 2017). The reason about the differences in results may be that the samples used in studies represent different phenotype classification of PD and came from different tissues.

Mitochondria DNA methylation levels may be reduced in ALS caused by superoxide dismutase-1 (SOD1) mutation. In postmortem ALS patients, DNMT1 and DNMT3A were observed at the nucleus and mitochondria of the motor cortex, and increased levels of DNMT3A protein were detected at the nuclear, soluble, and mitochondrial fractions (Chestnut et al., 2011). DNMT3A was also observed in mitochondria of adult mouse CNS, skeletal muscle and testis, and adult human cerebral cortex (Wong et al., 2013). The mitochondrial DNMT3A protein levels were significantly reduced in skeletal muscle and spinal cord at presymptomatic or early stage of disease in human *SOD1* transgenic mouse models of ALS (Wong et al., 2013). Subsequent studies reported that ALS patients, especially those with *SOD1* and Chromosome 9 Open Reading Frame 72 (*C9ORF72*) mutations, had an inverse correlation between the D-Loop methylation levels and mtDNA copy number (Stoccoro et al., 2018). However, only *SOD1* mutations resulted in a significant decrease in D-Loop methylation levels, suggesting that demethylation in the D-Loop region may represent a compensatory mechanism of mtDNA upregulation to counteract oxidative stress in ALS-linked *SOD1* mutation carriers (Stoccoro et al., 2018). The latest finding further confirmed this result. Patients with *SOD1* mutation and sporadic ALS patients showed lower levels of D-Loop methylation, while *C9ORF72*-ALS patients showed no significant difference in levels of D-Loop methylation compared with controls (Stoccoro et al., 2020). Therefore, the pattern of mtDNA methylation varies among diseases and mtDNA methylation levels may change along with the progression of the neurodegenerative diseases. However, further research is needed to identify the accurate mitochondrial gene methylation sites and elucidate their biological significance.

## Mitochondria Are Indirectly Involved in Histone Acetylation/Deacetylation via Acetyl-CoA and Nad^+^ in Neurodegenerative Diseases

### Acetyl-CoA Provided by Mitochondria Indirectly Affects Histone Acetylation and Benefits for AD Treatment

Histone acetyl transferases (Hats) transfer an acetyl group from acetyl-CoA to lysine ε-amino residues, which relaxes chromatin and increases the binding potential of transcriptional activators. Acetyl-CoA, the only donor of histone acetylation, is dynamically correlated with the acetylation levels of histones and transcription factors (Cai et al., 2011; Pietrocola et al., 2015; Shi and Tu, 2015). The acetyl-CoA levels are positively regulated by energy state in cells. When energy production is abundant, levels of acetyl-CoA are increased to promote histone acetylation and gene expression, and conversely, low energy reduces acetyl-CoA levels, thereby decreasing histone acetylation and inhibiting gene expression through chromatin concentration (Menzies et al., 2016).

Acetyl-CoA can be produced in cytoplasm, mitochondria and nucleus, and there are interactions between different pool of acetyl-COA. In mitochondria, acetyl-CoA is primarily synthesized by three pathways, including: (1) glycolysis; (2) β-oxidation of fatty acids (Rufer et al., 2009); and (3) the catabolism of branched amino acids (Harris et al., 2005). Besides, the mitochondrial enzyme acetyl-CoA synthetase short-chain family member 1 (ACSS1) can employ acetate to generate acetyl-CoA (Fujino et al., 2001; Figure 1). Acetyl-CoA synthesized in mitochondria cannot penetrate the mitochondrial membrane to reach the cytoplasm. Nevertheless, acetyl-CoA usually enters the TCA cycle to generate free CoA and citrate which can be exported from mitochondria via the mitochondrial tricarboxylate transporter (SLC25A1) and then catalyzed by ATP-citrate lyase (ACLY) to produce acetyl-CoA in cytosol and nucleus (Wellen et al., 2009; Zaidi et al., 2012; Pietrocola et al., 2015; Figure 1). Alternatively, the acetylcarnitine formed in the mitochondria is transported to cytoplasm by carnitine/acylcarnitine translocator and then enters the nucleus where acetylcarnitine is converted into acetyl-CoA by nuclear carnitine acetyltransferase (Madiraju et al., 2009; Figure 1). In cytosol and nucleus, acetyl-CoA can be generated from acetate by short-chain family member 2, a cytosolic counterpart of ACSS1 (Schug et al., 2015; Figure 1). Besides, acetyl-CoA synthesized in the cytoplasm can enter the nucleus directly (Figure 1).

The kinetics of histone acetylation largely depends on the concentration of acetyl-CoA, especially the ratio of acetyl-CoA to free CoA (Pietrocola et al., 2015). Studies suggest that the level of acetyl-CoA in mitochondria may influence histone acylation, though this requires direct evidence. For example, TP53 inducible glycolysis and apoptosis regulator (TIGAR), an endogenous inhibitor of glycolysis, was significantly increased during brain development as neural differentiation proceeding, especially in a rapid growth period of NSC differentiation (Zhou et al., 2019). Knocking out TIGAR could reduce the mRNA level of *ACLY* as well as acetyl-CoA production in mitochondria (Zhou et al., 2019). Simultaneously, levels of acetyl-CoA and H3K9 acetylation were also decreased at the promoter of NSC differentiation-related genes such as *Gfap*, *Neurod*, and *Ngn1* (Zhou et al., 2019). CMS12 and J147, two AD drug candidates, maintain mitochondrial homeostasis by regulating acetyl-CoA metabolism. They played a neuroprotective role by increasing acetyl-CoA production and increasing H3K9 acetylation in aging accelerated mouse tendency 8 (SAMP8) mice (Currais et al., 2019). Thus, increasing the level of acetyl-CoA can also be used as a drug target of inducing NSC differentiation and treating AD, but more evidence is needed to confirm it.

### NAD^+^ Provided by Mitochondria Indirectly Affects Histone Deacetylation Through Sirtuins

Histone deacetylases (HDACs) remove acetyl groups, resulting in chromatin contraction and gene transcription suppression. Sirtuins (SIRT1–SIRT7) are the only type of HDACs whose activities require NAD^+^ and are affected by the fluctuation of NAD^+^/NADH (Haigis and Sinclair, 2010). NAD^+^ is a cofactor of sirtuin deacetylase, which removes the acetyl group from the lysine residue of protein in an NAD^+^-dependent manner to produce nicotinamide (NAM) and acyl-ADP-ribose (Lautrup et al., 2019; Figure 1).

The sources of NAD^+^ in cells include diet, tryptophan synthesis and NAD^+^ depletion and recovery (Verdin, 2015; Lautrup et al., 2019). NAD^+^ distributes in the nucleus, cytoplasm and mitochondria, maintaining a constant balance between depletion and recycling. The cytoplasmic and nuclear NAD^+^ pools may be balanced by diffusion through the nuclear pore (Cantó et al., 2015). Since the mitochondrial membrane is impermeable to both NAD^+^ and NADH, a transport mechanism is required for their exchange between cytoplasmic and mitochondria (Stein and Imai, 2012). During glycolysis in cytoplasm, NAD^+^ is convert to NADH which is transferred to mitochondrial matrix via malate/aspartic acid shuttle and glyceraldehyde-3-phosphate shuttle (Madiraju et al., 2009; Figure 1). In mitochondria, NAD^+^ is reduced in the TCA cycle to produce multiple NADHs, and then NADHs are oxidized by complex I in ETC to produce NAD^+^ (Verdin, 2015; Figure 1). Although NAD^+^ is spread over in different compartments, the level of it in the cell may be limited (Pittelli et al., 2011). In yeast, malate-aspartic acid shuttle balances the NAD^+^/NADH ratio between the cytoplasm and the mitochondrial pool. The increase ratio of mitochondrial NAD^+^/NADH leads to the production of aspartic acid from malate via malate dehydrogenase (Mdh1) and asparagine (Aat1). Aspartic acid is transported to the cytoplasm via the AGC1 carrier, and then aspartic acid converted to malic acid by cytoplasmic malate dehydrogenase (Mdh2) and asparagine (Aat2), resulting in an increased cytoplasmic NAD^+^/NADH ratio (Easlon et al., 2008). It was also reported later that exogenous NAD^+^ could make the NAD^+^ level higher in mitochondria than in cytoplasmic, which indicated that the precursor or intermediate of NAD^+^ could penetrate the mitochondrial membrane (Pittelli et al., 2011). NR, the NAD^+^ precursor nicotinamide ribose, is likely to be converted into nicotinamide mononucleotide (NMN) in the cytoplasm, and NMN may pass through the mitochondrial membrane via nicotinamide mononucleotide adenosine transferase 1 (NMNAT) to produce NAD^+^ (Yang et al., 2007). It was interesting that NR increased proliferation and induces neurogenesis in the hippocampal dentate gyrus and the subventricular zones of aged mice (Zhang H. et al., 2016). Thus, mitochondria are one of NAD^+^ metabolism compartmentalization that may indirectly affect NAD^+^ concentrations in the nucleus and cytoplasm.

The sirtuins protein family is distributed in different compartments of the cells, including nucleus (SIRT1, SIRT6, and SIRT7), cytoplasm (SIRT2), and mitochondria (SIRT3-5; Haigis and Sinclair, 2010). SIRT1-3 showed strong deacetyl kinase activity, while the activities of SIRT4–7 were weak *in vitro* (Michishita et al., 2005). SIRT1 and SIRT2 could acetylate histones or non-histone proteins, and they were able to travel between nucleus and cytoplasm, while SIRT3 primarily performed post-translational modification of proteins in mitochondria (Tanno et al., 2007; Jing and Lin, 2015). SIRT1 and SIRT2 were the most abundant sirtuins in cultured cells isolated from normal adult brain tissues (Jayasena et al., 2016). The expression level of SIRT1 was the highest in neurons, and SIRT2 was highly enriched in adult human frontal lobes (Jayasena et al., 2016).

### SIRT1 and SIRT2 Play an Opposite Role in PD Under Mitochondria Dysfunction

In the PD cell model induced by rotenone, an inhibition of respiratory chain complex I, SIRT1 bound to H3K9 in the p53 promoter region, resulting in decreased H3K9 acetylation and increased H3K9 trimethylation, thereby inhibiting p53 gene transcription and reducing rotenone-induced apoptosis (Feng et al., 2015). 1-methyl-4-phenylpyridinium (MPP^+^) is also an inhibitor of respiratory chain complex I, which may induce ROS production and increase HIF-1α expression in SH-SY5Y cells. Inhibiting SIRT1 expression could significantly increase H3K14 acetylation in the HIF-1α promoter region, leading to transcriptional activation (Dong et al., 2016). It was found in earlier years that the direct binding of α-synuclein to histones, which reduced the level of histone H3 acetylation in cultured cells and maybe cause the nuclear toxicity of α-synuclein (Kontopoulos et al., 2006). Further study demonstrated that HDAC inhibitor of SIRT2 could save the toxicity of α-synuclein in a PD cell model (Outeiro et al., 2007). A similar phenomenon was observed that oxidative stress induced the relocalization of α-synuclein into nucleus (Siddiqui et al., 2012). Then α-synuclein subsequently bound to the PGC1-α promoter, which resulted in histone deacetylation, thereby reducing the expression of PGC1-α and impaired mitochondrial function (Siddiqui et al., 2012). Therefore, in PD, increasing SIRT1 can repair mitochondrial dysfunction and plays a neuroprotective role. On the other hand, inhibiting SIRT2 can alleviate the toxicity of α-synuclein and may enhance mitochondrial function.

## Non-Coding RNAs Are Involved in Neurodegenerative Diseases by Regulation Mitochondria Functions

**FIGURE 3:**
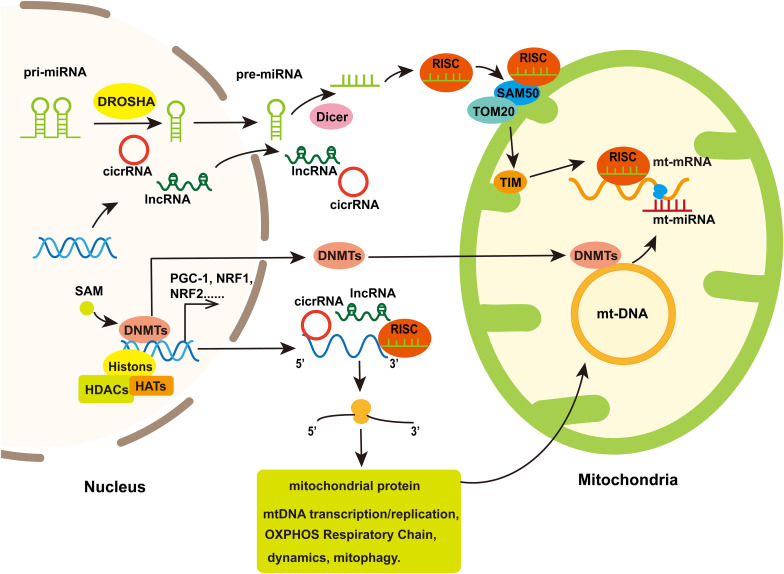
The involvement of mitochondria in nuclear epigenetic regulation and mtDNA methylation. Mitochondrial genomes can be modified by methyltransferases which are transport from nuclear to mitochondria. Simultaneously, Histone acetylation/deacetylation and DNA methylation participate in the expression of nuclear-encoded genes regulated mitochondrial function including some transcription factor that regulated genes associated with mitochondrial biogenesis. Non-coding RNA, including miRNA, lncRNA, and cicrRNA, coded by nucleus genome or several miRNAs coded by mt-DNA, can regulate genes encoding part of proteins in mitochondria to influence mitochondria biogenesis, mitochondrial dynamics, mitophagy, and cell apoptosis. In addition, the nuclear-encoded miRNAs assemble with Ago to form the RNA-induced silencing complex (RISC), which is improved into the mitochondria through protein channels in mitochondria membrane and thereby regulate mt-mRNA translation.

MiRNA, siRNA, piRNA, lncRNA, and circRNA are non-coding RNAs involved in epigenetic regulation (Panni et al., 2020). In mitochondria, non-coding RNAs are derived from nuclear or mitochondria genome. However, mitochondrial-encoded non-coding RNAs are rarely reported in the study of neurodegenerative diseases. And the process about how nuclear-encoded non-coding RNAs enter into mitochondria is unclear beside miRNA. One of hypotheses about how miRNAs enter into mitochondria is that the complex of AGO2 and miRNA crosses the OMM via SAM50 and TOM20 and then they are translocated into mitochondrial matrix through the IMM (Macgregor-Das and Das, 2018; Figure 3). Several miRNAs were found altered in mitochondrial fractions of hippocampal tissue after controlled cortical impact injury in rats, which suggested the regulation of mitochondrial miRNAs to cerebral nerve injury (Wang et al., 2015, 2021). It implies the possibility of mitochondrial miRNAs regulating neurodegenerative diseases, but the related research is still sparse.

The regulation of nuclear-encoded non-coding RNAs on mitochondrial function plays a crucial role in the onset or treatment of neurodegenerative diseases (Wu and Kuo, 2020). In this review, we will focus on the regulatory mechanisms of non-coding RNAs on mitochondrial homeostasis in neurodegenerative diseases.

### MiRNAs Regulate Neurodegenerative Diseases by Targeting Genes Associated With Mitochondria Functions

MiRNAs are short non-coding RNAs regulating gene expression at post-transcription level. In cytoplasm, miRNAs, after being processed by Dicer, interact with Argonaute (AGO) proteins and assemble into RNA-induced silencing complex and then bind to the 3′ UTR region of mRNA to promote mRNA degradation or inhibit protein translation (Fabian and Sonenberg, 2012; Figure 3). MiRNAs can be transported between various cell compartments such as nucleus, cytoplasm and mitochondria to regulate the target mRNA translation or even transcriptional rate (Makarova et al., 2016). In addition, miRNAs can be secreted extracellularly as signaling molecules to mediate intercellular communication (O’Brien et al., 2018).

MiRNAs targeting genes associated with mitochondria biogenesis and ECT activity have neuroprotective effects on diseases and provide potential therapeutic strategies for patients. The up-regulated expression of miR-590-3p and miR-144-3p in AD and PD disease models, respectively, to improve mitochondrial function by increasing the expression levels of PGC-1α, NRF1, and TFAM (Li et al., 2016; Wang J. et al., 2016). In AD, the innate immune system activated by oligomeric amyloid β42 (oAβ42) was likely to trigger chronic neuroinflammation (Salminen et al., 2009). *In vitro* study showed that oAβ42 stimulated the production of TNF-α in mitochondria, increased the expression level of miR-34a, and reduced the expression of five key proteins in the mitochondrial ETC, including NDUFC2 in complex I, SDHC in complex II, UQCRB and UQCRQ in complex III, COX10 in complex V (Russell and N Doll, 2016). 11 miRNAs families in the frontal cortex of PD patients might influence the mitochondrial biogenesis by potentially targeting *PGC1-α* or its upstream regulators (Thomas et al., 2012). In ALS, miR-23a was discovered to inhibit the activity of PGC1-α in skeletal muscle (Russell et al., 2013).

Increasing mitochondrial fusion has a protective effect on the onset and escalation of neurodegenerative diseases. The expression of MFN2 was decreased in the hippocampus and cortical neurons of AD patients (Manczak et al., 2011; Wang H. et al., 2019). In a phenotypic SAMP8 model similar to the symptoms of late-onset and age-related sporadic AD patients, miR-195 targeted and inhibited *MFN2* expression in the mice hippocampus, reduced the MMP and caused mitochondrial dysfunction (Zhang R. et al., 2016). Another study also found that increasing mitochondrial fusion was beneficial to preventing AD. Mutations in the APP are able to trigger AD. In APP mutant cells, the mRNA and protein levels of mitochondrial biogenesis (PGC1-α, NRF1, NRF2, and TFAM) and synaptic genes (synaptophysin and PSD95) were reduced, and upregulating of miR-455-3p could rescued the decreased expression of these genes (Kumar et al., 2019). In HD patients, the protein levels of DRP1 and FIS1 were increased while the protein levels of MFN1, MFN2, OPA1, and TOMM40 were decreased (Shirendeb et al., 2011). Exogenous expression of miR-214 could inhibit MFN2 expression, increase mitochondrial fragment distribution, and alter cell distribution at different stages of the cell cycle, which might interfere with the pathogenesis of HD (Bucha et al., 2015).

MiRNA can participate in regulating mitophagy to maintain the mitochondrial quality. Mutations in *PINK1* and *Parkin* are the common cause of autosomal recessive PD (Pickrell and Youle, 2015). miR-27a/b could target *PINK1* to decrease its translational level, which led to the inhibition of the accumulation of PINK1, parkin transportation and the expression of LC3II after mitochondrial injury (Kim et al., 2016). Simultaneously, autophagy of lysosomal clearance in damaged mitochondria was inhibited (Kim et al., 2016). In the PD mouse model and SH-SY5Y cell model, the expression levels of miR-103A-3p were increased, which inhibited *Parkin* expression and the clearance of damaged mitochondria (Zhou et al., 2020). Rat treated with rotenone resulted in an oxidative imbalance in the brain and activation of NF-Kβ. Activated NF-Kβ induced miR-146a transcription by banding to miR-146a promoter region, thereby downregulating Parkin protein levels and causing mitochondrial damage and dysfunction (Jauhari et al., 2020).

The damaged mitochondria undergo autophagy and eventually degradation in the lysosome, which is a key to control mitochondrial quality. MiR-5701 was able to reduce the mRNA levels of genes involved in lysosomal biogenesis and mitochondrial quality control, such as *VCP*, *LAPTM4a*, and *ATP6V0D1* (Prajapati et al., 2018). The decreased expression of those gene led to mitochondrial dysfunction, defective autophagy flux and further made SH-SY5Y cells sensitive to the neurotoxin 6-hydroxydopamine (6-OHDA) -induced cell death (Prajapati et al., 2018).

MiRNAs are involved in regulating ROS-induced apoptosis in neurodegenerative diseases. Bax is a proapoptotic member of BCl-2 family. The production of ROS resulted in the formation of pores at mitochondrial membranes, which could recruit Bax, promote the release of Cyt c, and activate caspase-mediated cascade amplification reaction to induce apoptosis of mitochondrial pathway (Orrenius et al., 2007; Sinha et al., 2013). However, antiapoptotic proteins of Bcl-2 family such as Bcl-2 and Bcl-X_L_ inhibited the activity of these proapoptotic proteins by preventing oligomerization (Orrenius et al., 2007). MiR-7 and miR-153 were able to regulate mitochondrial ROS-mediated α-synuclein protein synthesis and reduce MPP ^+^ -mediated α-synuclein level (Je and Kim, 2017). MiR-7 overexpression inhibited the release of ROS and Cyt c responding to MPP^+^ in human neuroblastoma SH-SY5Y cells (Chaudhuri et al., 2016). Bim enhanced Bax mitochondrial translocation by activating JNK/c-Jun, which induced Cyt c release and led to the apoptosis of dopaminergic neurons (Perier et al., 2007). MiR-124 was able to suppress Bax translocation to mitochondria by inhibiting Bim in MPTP-treated mice. Morever, upregulating the expression of miR-124 could alleviate the characteristics of MPP^+^-intoxicated SH-SY5Y cells, such as impaired autophagy process, autophagosome accumulation and lysosomal depletion (Wang H. et al., 2016). In the PD cell model induced by 6-OHDA, inhibiting miR-410 reduced the viability of neuronal cells and increased capase-3 activity, ROS production and apoptosis (Ge et al., 2019).

### LncRNAs and CircRNAs Regulate Neurodegenerative Diseases by Targeting Genes Associated With Mitochondria Functions

In addition to miRNAs, some lncRNAs and circRNAs have also been found to regulate mitochondrial function and play a role in the progression of neurodegenerative diseases. LncRNAs is long non-coding RNAs that directly interact with transcription factors, functional RNA and chromatin remodeling modifiers to regulate gene expression at the transcriptional, post-transcriptional and epigenetic levels, respectively, (Kopp and Mendell, 2018). CircRNAs are characterized by covalently closed loop structures. They can act as miRNA sponges to reduce the inhibition of miRNAs to target genes (Du et al., 2017).

In PD studies, a decrease in the level of lncRNA AL049437 was able to increase cell viability, MMP, mitochondrial mass, and tyrosine hydroxylase secretion, on the contrary, knocking out AK021630 had the opposite effect (Ni et al., 2017). Hence, it was speculated that lncRNA AL049437 may cause the risk of PD, and lncRNA AK021630 might inhibit the development of PD (Ni et al., 2017). LncRNA MALAT1 was highly expressed in the brains of MPTP-induced PD mouse model and LPS/ATP-induced mouse BV2 microglia. Knockdown of MALAT1 inhibited the expression of NRF2, thereby inhibiting inflammasome activation and ROS production (Cai et al., 2020). The expression of circDLGAP4 was reduced both in the MPTP-induced PD mouse model and MPP^+^-induced PD cell model (Feng et al., 2020). *In vitro* study have shown that circDLGAP4 promoted cell viability, reduced apoptosis, mitochondrial damage, and enhanced autophagy, which reduced the neurotoxic effect of MPP ^+^ in SH-SY5Y and MN9D cells (Feng et al., 2020).

In Aβ_25__–__35_-induced AD model of PC12 cells, the silence long non-coding RNA brain-derived neurotrophic factor anti-sense inhibited Aβ_25__–__35_-induced apoptosis by inhibiting the release of Cyt c, which increased the expression of Bcl-2 and reduced the expression of caspase-3 and Bax (Guo et al., 2018). In SH-SY5Y and HPN cells, lncRNA SNHG15 reduction partially rescued the effects of Aβ_25__–__35_ treatment on cell viability, apoptosis, MMP, caspase-3 activity and apoptosis-related protein levels (Wang H. et al., 2019). LncRNA NEAT1 with elevated expression level interacted with NEDD4L and promoted the ubiquitination of PINK1 to impair PINK1-dependent autophagy in animal model of AD (Huang et al., 2020). Another research in AD showed that overexpression of lncRNA WT-AS inhibited the expression of transcription factor WT which suppressed the expression of miR-375 and SIX4 (Wang et al., 2020). And lncRNA WT-AS could inhibit the pTau protein and promote the production of ATP and therefore play a role in the regulation of mitochondrial structure and function (Wang et al., 2020).

## The Involvement of Mitochondria in Epigenetic Regulation Can Provide New Strategies for Neurodegenerative Diseases Treatment

Treatment or diagnosis of diseases based on epigenetic regulation included epigenetic modified biomarkers, chemical drugs and miRNA-targeting drugs (mimics or inhibitors). The causes of majority of neurodegenerative diseases are hereditary, thus pathogenic genes or single nucleotide polymorphisms can be used as early diagnostic markers (Gotovac et al., 2014). In addition, neurodegenerative diseases have sporadic cases, and the influence of environmental factors on the onset of diseases cannot be ignored. Therefore, epigenetic biomarkers have attracted attention. Many studies on DNA methylation markers of neurodegenerative diseases have been recognized (Fetahu et al., 2019; Wang C. et al., 2019; Vasanthakumar et al., 2020). Currently, mitochondria have attracted increasing attention in the field of epigenetics. Fluctuations in mtDNA methylation levels have been observed in AD, PD, and ALS compared with normal population, suggesting that mtDNA methylation studies has therapeutic potential in neurodegenerative diseases. However, due to the lack of functional studies on mtDNA methylation, it has not entered the clinical application stage.

The effects of drugs and compounds targeting mitochondria of neurodegenerative diseases are mainly antioxidant, improving mitochondrial biogenesis and increasing energy generation (Stanga et al., 2020). Some drugs targeting DNA methylation have been shown to be effective on mtDNA. For instance, valproic acid, a histone deacetylase inhibitor, is an anticonvulsant and mood stabilizer and a pharmacological tool used in the study of nuclear epigenetics, such as DNA methylation. After few days of treatment with valproic acid in mouse 3T3-L1 cells, it was observed a decrease in 5hmC levels of mtDNA, while 5mC level was not affected (Chen et al., 2012). In addition to drugs, overall methylation levels can be improved by regulating nutritional supplement. For example, One-C metabolism in mitochondria can regulate the production of SAM, and external nutrients such as vitamin B12 and folic acid can promote the production of SAM and thus enhance DNA methylation (Li et al., 2015; Román et al., 2019). Moreover, NAD^+^ and NR are used to treat AD and ALS. Their main role is to enhance mitochondrial biogenesis and inhibit ROS (Chaturvedi et al., 2009; Li et al., 2015; Cenini and Voos, 2019; Carrera-Juliá et al., 2020). NAD^+^ is a co-regulator of histone acetylation. Whether exogenous NAD^+^ can improve disease by regulating histone acetylation remains to be studied. Similarly, acetyl-CoA, a metabolic intermediate of mitochondria, is the acetyl group donor for histone acetylation. At present, there are two AD drugs that can enhance the methylation of histone H3K9 by increasing the level of acetyl-CoA, maintain mitochondrial homeostasis and play a neuroprotective role (Currais et al., 2019).

Since miRNAs can regulate the expression of endogenous genes and biological pathways, taking miRNA as a target for the treatment of diseases has become a very interesting research direction (Junn and Mouradian, 2012). Anti-miRNA drugs or miRNA analogs can be delivered to the body as a personalized therapy using viral vectors, lipids, nanoparticles, and exosomes (Lakhal and Wood, 2011; Zhao et al., 2016; Paul et al., 2020). However, there are still many problems to be solved. As more and more miRNAs have been shown to improve disease traits by regulating mitochondrial function, it is likely that miRNA will become a targeted therapeutic strategy in the future.

In general, although the involvement of mitochondria in epigenetic regulation have provided the potential strategies for neurodegenerative diseases, more in-depth studies are required to research and development of mitochondria-specific drugs.

## Conclusion and Future Perspectives

**FIGURE 4:**
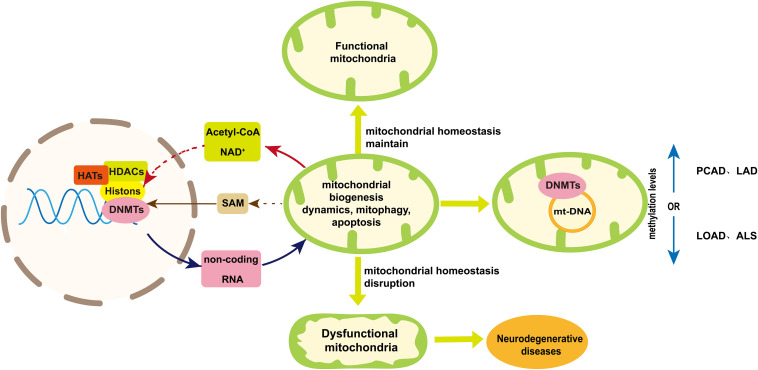
A schematic diagram of mtDNA methylation and the bidirectional communication between mitochondria and nuclear in epigenetic regulation. Mitochondria maintain their homeostasis in several ways including mitochondrial biogenesis, dynamic, mitophagy and apoptosis. Disruption of mitochondrial homeostasis will result in impaired mitochondrial function, which may contribute to neurodegenerative diseases. Mitochondria are involved in the epigenetic regulation from two aspects. One is mtDNA methylation, the extent of which varies among neurodegenerative diseases. The other is the bidirectional communication between mitochondria and nuclear, which is essential to maintain mitochondria homeostasis and can play a role in nuclear epigenetic regulation of neurogenerative diseases. Acetyl-CoA and NAD^+^ provided by mitochondria indirectly play a role in histone acetylation and deacetylation, respectively, to influencing mitochondria homeostasis. Mitochondria can influence the production of SAM that regulated nuclear DNA methylation. On the other hand, non-coding RNAs coded by nuclear genome can regulate neurogenerative diseases by influencing mitochondria homeostasis. Dashed arrows indicate pathways that need further validation. Abbreviations: PCAD, preclinical Alzheimer’s disease; LAD, late-stage Alzheimer’s disease; LOAD late-onset Alzheimer’s disease; ALS, amyotrophic lateral sclerosis; and SAM, S-adenosyl methionine.

In this review, we discuss the role of mtDNA, metabolic intermediate (SAM, acetyl-CoA and NAD^+^) and non-coding RNAs in the epigenetic regulation of neurodegenerative diseases (Figure 4). The regulation of nuclear-encoded non-coding RNAs is able to improve neurodegenerative diseases by enhancing mitochondria biogenesis, maintaining mitochondria quality and reducing apoptosis (Je and Kim, 2017; Ni et al., 2017; Kumar et al., 2019; Feng et al., 2020; Zhou et al., 2020). On the other hand, the extent of mtDNA methylation varies among neurodegenerative diseases (Blanch et al., 2016; Stoccoro et al., 2020). But the mechanism of mitochondrial DNA methylation in neurodegenerative diseases requires further study. Available literatures indicate acetyl-CoA and NAD^+^ provided by mitochondria may indirectly affect the histone acetylation and deacetylation, respectively, in the research of neurodegenerative diseases. The level of SAM can be increase by taking vitamin B12 and folic acid to enhance the methylation level for neurodegenerative disease treatment. However, the sources of acyl-CoA and NAD^+^ participated in histone modification are needed to be tracked to elucidate the role of mitochondrial metabolites involved in epigenetic regulation (Trefely et al., 2020). And a deeper understanding of the relationship between mitochondrial metabolism and epigenetics in neurodegenerative diseases is required in order to provide new treatment strategies.

## Author Contributions

SX, YL, and SL conceived the manuscript. SX, XZ, and CL wrote the manuscript. QL and HC contributed to crafting figures. YL and SL reviewed and edited the manuscript. All authors listed have made a substantial, direct and intellectual contribution to work, and approved it for publication.

## Conflict of Interest

The authors declare that the research was conducted in the absence of any commercial or financial relationships that could be construed as a potential conflict of interest.

## Publisher’s Note

All claims expressed in this article are solely those of the authors and do not necessarily represent those of their affiliated organizations, or those of the publisher, the editors and the reviewers. Any product that may be evaluated in this article, or claim that may be made by its manufacturer, is not guaranteed or endorsed by the publisher.
